# Scaling of joint mass and metabolism fluctuations in in silico cell-laden spheroids

**DOI:** 10.1073/pnas.2025211118

**Published:** 2021-09-15

**Authors:** Ermes Botte, Francesco Biagini, Chiara Magliaro, Andrea Rinaldo, Amos Maritan, Arti Ahluwalia

**Affiliations:** ^a^Research Centre “E. Piaggio,” University of Pisa, 56122 Pisa, Italy;; ^b^Department of Information Engineering, University of Pisa, 56126 Pisa, Italy;; ^c^Laboratory of Ecohydrology, École Polytechnique Fédérale de Lausanne, 1015 Lausanne, Switzerland;; ^d^Department of Civil, Environmental, and Architectural Engineering, University of Padova, 35122 Padova, Italy;; ^e^Department of Physics and Astronomy “G. Galilei,” University of Padova, 35122 Padova, Italy

**Keywords:** finite element models, spheroids, scaling, covariations

## Abstract

Variations and fluctuations are characteristic features of biological systems and are also manifested in cell cultures. Here, we describe a computational pipeline for identifying the range of three-dimensional (3D) cell-aggregate sizes in which nonisometric scaling emerges in the presence of joint mass and metabolic rate fluctuations. The 3D cell-laden spheroids with size and single-cell metabolic rates described by probability density functions were randomly generated in silico. The distributions of the resulting metabolic rates of the spheroids were computed by modeling oxygen diffusion and reaction. Then, a method for estimating scaling exponents of correlated variables through statistically significant data collapse of joint probability distributions was developed. The method was used to identify a physiologically relevant range of spheroid sizes, where both nonisometric scaling and a minimum oxygen concentration (0.04 mol⋅m^−3^) is maintained. The in silico pipeline described enables the prediction of the number of experiments needed for an acceptable collapse and, thus, a consistent estimate of scaling parameters. Using the pipeline, we also show that scaling exponents may be significantly different in the presence of joint mass and metabolic-rate variations typically found in cells. Our study highlights the importance of incorporating fluctuations and variability in size and metabolic rates when estimating scaling exponents. It also suggests the need for taking into account their covariations for better understanding and interpreting experimental observations both in vitro and in vivo and brings insights for the design of more predictive and physiologically relevant in vitro models.

## Significance

Allometric scaling has many applications, from the prediction of pharmacokinetics in animals and humans to the probing of ecosystem dynamics. Most studies have neglected to account for variations and fluctuations, although they are intrinsic features of all biological systems. To understand how metabolic scaling emerges in the presence of variations, we developed computer-generated models of cell-laden spheroids to define the experimental size range of cell cultures with quantifiable similitudes in terms of fluctuations and metabolic scaling with living organisms. We show that the estimates of scaling exponents may change with increasing variability in both mass and metabolic rate. The computational pipeline described underpins the sound design of statistically meaningful cell-based models, with impacts in both biomedical science and ecology.

Allometric scaling behavior is characteristic of all living organisms (1). The most well-known scaling theory is Kleiber’s law (KL), which states that the metabolic rate (*B*) of an organism scales to the 3/4 power of its mass (2). KL forms the basis of several ecological and biological studies, despite the intense debate on its significance and theoretical origins (3–7) and on the precise value of the mass–*B* scaling coefficient (8–10).

Scaling laws have also been viewed with interest in the field of tissue engineering and biotechnology (11–13). Given that metabolism is a fundamental biological function, KL is thought to represent a benchmark for physiological relevance in in vitro–engineered constructs, which are designed to resemble the structural and functional features of a tissue or organ (14, 15). Some studies have suggested that the behavior of cells in vivo is different from in vitro, switching from an allometric to an isometric behavior (16, 17). West et al. (16) surmise that the number of mitochondria in any cell is constant when in culture, resulting in a higher oxygen-consumption rate with respect to the in vivo state. Glazier (17) suggests that, in addition to energetic and physical constraints, systemic regulation can be crucial for the emergence of allometric behavior.

Much of the data they use have been extrapolated from two-dimensional (2D) monolayer cultures, where all the cells are exposed to the same amount of oxygen. In recent decades, 2D culture techniques have been replaced by three-dimensional (3D) methods, culminating—in the last few years—in the development of organoids. Based on these novel approaches, a number of investigations have shown that oxygen-consumption rates are lower in 3D cell aggregates than in 2D monolayers (18–20). Moreover, recent computational studies demonstrate that 3D aggregates with high cell densities (i.e., spheroids and organoids) can obey KL in a specific mass range, even in the absence of a resource-supplying network (14, 21).

It should be noted that most reports in the literature dealing with scaling laws in biology—be they field studies or laboratory investigations—consider the average values of the individual quantities involved (e.g., mass and *B*) (22, 23). There are, for instance, a number of excellent studies on the analysis of shifts in scaling exponents in an evolutionary perspective or the identification of biophysical constraints underpinning the isometry to nonisometry transition (24–26). However, this typically deterministic approach does not take into account the heterogeneities of living organisms, which are characterized by biological noise (27)—intrinsic and extrinsic fluctuations due to thermodynamics, genetic diversity, resource availability, etc.—which may also underlie the debate around KL. Fluctuations in cells and cellular processes are well known and play an important role in the origin of variability in organisms (27, 28). Although usually attributed to extrinsic parameters, it is widely recognized that organoids and spheroids display variability. The experimental observations might, at least in part, be the result of variability in their size, as well as of the intrinsic stochasticity of enzyme-mediated metabolic reactions. Rather than being discarded or considered a nuisance, fluctuations may prove relevant to interpret empirical observations, judge the reliability of predictions, and understand the dynamics of organoid sensitivity to external perturbations (29–31). They could, for instance, shed light on the determinants of metabolic scaling and provide the means for testing competing models and explanations. However, several data points are required to properly characterize distributions, with inevitably high time and economic costs (31). Moreover, measuring the variability of microscaled systems is a technical challenge, as in many cases, the resolution of sensors for measurements of *B* and size or mass may be in the same range of the intrinsic variations (32).

There have been some attempts to develop theoretical and methodological frameworks to evaluate the power-law relationship between mass and *B* in the presence of fluctuations. For instance, Giometto et al. (30) showed how masses of different species of protists can be described by a single universal distribution curve according to a theoretical finite-size scaling framework. Their model implies that the variance of mass of a species increases quadratically with its mean value. Thus, the intrinsic variance of the species can impact the scaling of other dependent variables, such as *B*. Zaoli et al. (31) took the framework further and demonstrated that probability distributions of nutrient uptake rates (a proxy of *B*) of different species of freshwater phytoplankton could again collapse onto a generalized scaling function. In the absence of sufficient experimental data, both studies determined only marginal distributions and were limited in the number of experimental samples, and Zaoli et al. were particularly challenged by the difficulty in precisely measuring the *B* of single phytoplankton within a population. Taken together, the experimental evidence—in the form of consistent marginals gathered in refs. 30 and 31—suggests the existence of a generalized scaling functional describing the joint mass–*B* probability distribution, as hypothesized by Zaoli et al. (31).

Based on these premises, spherical cell aggregates with random fluctuations in mass and single-cell oxygen-consumption rates (sOCRs) were generated *“*in silico,” simulating laboratory experiments that, as discussed above, may be unfeasible with current technologies. The aim was to develop a computational, or in silico, pipeline for supporting investigations on fluctuations and metabolic scaling in cell aggregates in vitro. The pipeline was used to identify the range of sizes in which cell aggregates can be considered physiologically relevant, defined here as those with less than 10% of their volume below a threshold oxygen concentration for viability, and that manifest a scaling exponent significantly different from one, following the generalized scaling functional proposed in Zaoli et al. (31). It was further used to explore how scaling exponents may differ as the amplitude of mass and metabolic-rate variations change.

First, by using finite element (FE) methods, the oxygen profiles and metabolic rates were computed in 3D cell-laden spheroids spanning four orders of magnitude in mass. Then, the mass and *B* distributions were rescaled, and optimization procedures were used to identify the scaling exponents that best collapsed those distributions, according to the formulation in ref. 31. In particular, as numerical methods for collapsing joint probability density functions have not been reported, we developed a method for collapsing multivariate distributions. We were thus able to characterize the scaling behavior of spheroids in the presence of variability of both parameters. The large number of cell-laden spheroids generated also allowed the identification of the minimum number of samples for obtaining consistent collapses of joint mass–*B* probability distributions across scales. The results can be used to design physiologically relevant in vitro models, taking into account the intrinsic fluctuations in biological systems.

## Methods

### Computational and Statistical Analysis.

**Fig. 1.**
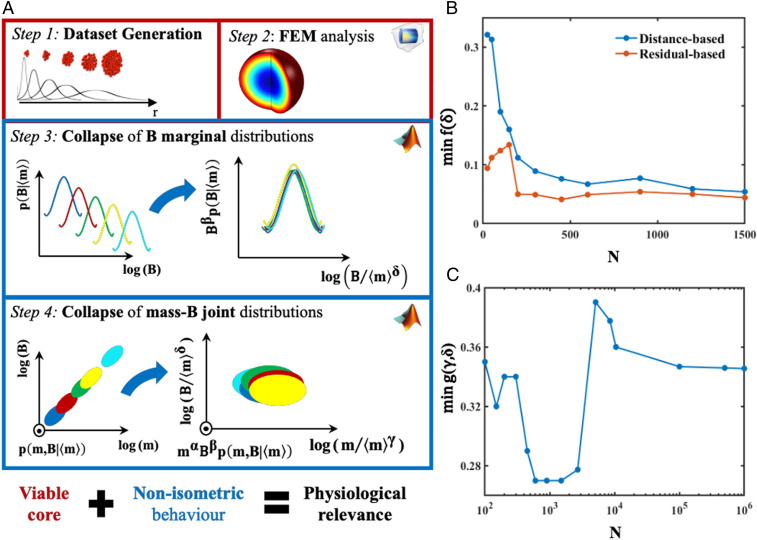
(*A*) In silico pipeline for the identification of a physiologically relevant size window through the analysis of size-dependent scaling in 3D cell aggregates, taking into account fluctuations in mass and metabolic rates. FEM, FE method. (*B*) Minima of *f*^*res*^(*δ*) (orange) and of *f*^*dis*^(*δ*) (blue) against the number of samples *N* per spheroid size distribution: an example for three collapsing distributions (from the 9th to the 11th; *SI Appendix*, Table S2). A stable minimum is reached at *N* = 200. (*C*) Minima of *g*(*γ*, *δ*) against the number of samples *N* per spheroid size distribution: an example for three collapsing distributions (from the 9th to the 11th). The plateau, indicating a stable minimum, is at *N* = 10^4^. For the sake of clarity, the *N* axis is plotted on a log scale.

The computational and statistical analysis pipeline is illustrated in Fig. 1*A*. Data generation and statistical analyses were performed by using MATLAB (The MathWorks Inc.) routines, and the FE simulations were performed by using Comsol Multiphysics (version 5.4; COMSOL AB).

### In Silico Data Generation.

In general, microorganism and cell volumes are log-normally distributed (30). Based on experiments and literature data on cell and spheroid populations, we generated cell-laden 3D spheroids (referred to in the text simply as spheroids) with log-normally distributed radii, *R*, gathered around 17 mean values, ⟨*R*⟩, ranging from 31 to 5,000 μm (14, 21). The methods and experimental datasets are reported in *SI Appendix*, Fig. S1, Tables S1 and S2. The *k*th-order moment was defined as follows (30, 31):

$$\langle R^{k}\rangle \;=q_{k}{\langle R\rangle }^{k},$$ [1]

where *q*_*k*_ is a factor of proportionality. As demonstrated in ref. 30, Eq. **1** is an inherent property due to the log-normality of size distributions. On the basis of our experimental data, the second-order coefficient was set to *q*_*2*_ = 0.01 (*SI Appendix, *Table S1). Starting from *N* = 25 up to *N* = 10^6^, spheroid radii were randomly generated for each of the 17 size distributions (*SI Appendix*, Table S2).

The average mass of each size distribution was derived from the average radius as ⟨*m*⟩ = ω*(4/3)*π* ⟨*R*⟩^3^, where the density of the 3D spheroid, *ω* (kg⋅m^−3^), was approximated to be homogeneous, isotropic, and equal to that of the water.

### FE Simulations.

FE models of the reaction–diffusion equation, coupling Fick’s law and Michaelis–Menten kinetics, were implemented considering spherical symmetry (i.e., only depending on the radial coordinate *r*):

$$\frac{\partial c}{\partial t}=D\left(\frac{1}{r^{2}}\right)\frac{\partial }{\partial r}\left(r^{2}\frac{\partial c}{\partial r}\right)-V_{M}\;\left(c,\;sOCR\right)\;\text{,}$$ [2]

where *c* is the oxygen concentration (mol⋅m^−3^), *D* is the diffusion constant of oxygen in water (m^2^⋅s^−1^), and *V*_*M*_ is the intrinsic volumetric oxygen-consumption rate of the whole cell-laden spheroid, which is assumed to be governed by Michaelis–Menten kinetics (33).

$$V_{M}\left(c,\;sOCR\right)=\;\frac{sOCR\;\rho_{c}\;c}{k_{M}\;+\;c}.$$ [3]

Here, the sOCR is in mol⋅s^−1^⋅cell^−1^, *ρ*_*c*_ is the cellular density (cells⋅m^−3^), and *k*_*M*_ (mol⋅m^−3^) is the Michaelis–Menten constant.

Two different cell phenotypes—stem cells (21) and hepatocytes (14)—with different mean values of sOCR were considered, while *ρ*_*c*_ and *k*_*M*_ were maintained constant. Besides variability in size, we also considered variability in cell metabolism due to aleatory factors [e.g., thermodynamic effects or biochemical constraints (16, 17)]. Based on the data reported by Wagner et al. (34), a Gaussian distribution with a relatively small SD (20% of the mean value) was attributed to the sOCR. Using a MATLAB routine, we generated up to 10^6^ sOCR values with a Gaussian distribution, randomly pairing them with the *N* spheroid radii within the size distributions. All the parameters used are listed in *SI Appendix*, Table S3.

### Extraction of the Metabolic Rates and the Nonviable Core Volumes.

After generating the mesh using the predefined “extrafine” mesh size, the models were solved in stationary conditions.

The results of the FE simulations were then postprocessed for estimating the metabolic rate (*B*) as the magnitude of the inward flux at the spheroid surface multiplied by its total surface area (14).

$$B\;=\;D{\frac{\partial c}{\partial r}}_{r=R}*4\pi R^{2}\;.$$ [4]

As the core of 3D cellular aggregates is often deprived of oxygen (21, 35), we also determined the volume of the spheroid at which the oxygen concentration is below a critical threshold for viability, *C*_crit_ = 0.04 mol⋅m^−3^ (36), expressing it as a percentage of the total volume (% nonviable volume, Φ). Spheroids with nonviable cores occupying more than 10% of their total volume were considered as not physiologically relevant.

### Study of *B* Marginal Distributions.

Prior to studying joint mass–*B* distributions, we established a rigorous procedure for evaluating the existence of a general probability density function describing the *B* marginal distributions and determining its size-dependent scaling law.

In particular, as in Zaoli et al. (31), the marginal probability density function was assumed to have the following scalable form:

$$p\left(B|\left\langle m\right\rangle \;,\;\beta \right)=\;B^{-\beta }F\left(\frac{B}{\langle m\rangle^{\delta }}\right)$$ [5]

where *β* is a normalization exponent, *δ* is the scaling exponent defining the relation between sizes (or masses; *m*) and metabolic rates (*B*), and *F* is a general scaling function. *β* was kept equal to one, consistent with the constraint derived in ref. 30, which links the normalization and scaling exponents through the application of the first-order moment (i.e., mean value, ⟨*m*⟩) definition of Eq. **5**.

To identify the best collapse, a suitable functional for quantifying the closeness of three or more adjacent size distributions after rescaling with respect to ⟨*m*⟩^*δ*^ must first be defined. Current methods for quantifying the statistical distance between probability distributions [e.g., Hellinger’s distance or Battacharjee and Seno’s method (37, 38)] leverage on the concept of probability contiguity, assuming the existence of colinear points. They work well for optimizing the collapse of marginal distributions, but are particularly challenging for maximizing the overlap of surfaces in a 3D space starting from joint-frequency histograms. Indeed, as far as we know, methods for collapsing joint probability density functions and for assessing whether they belong to a common distribution have not been reported. We, therefore, developed a functional for optimizing marginal and joint distribution collapse without resorting to interpolation or relying on the a priori knowledge of the functional form of probability distributions. Our method is denoted as the “distance-based” method—with a minimization functional *f*^*dis*^(*δ*)—and basically entails minimizing the sum of the squares of the distance between distributions while displacing them in the rescaled mass–*B* plane. The computational performance and collapse accuracy of the distance-based method was compared with Battacharjee and Seno’s “residual-based” method—characterized by the functional *f*^*res*^(*δ*)—(38). A more comprehensive rationale and theoretical details on the two methods can be found in *SI Appendix*.

An iterative process (summarized in *SI Appendix*, Fig. S2) was used to group the size distributions into “size windows,” which effectively collapse with a common scaling coefficient *δ*. Briefly, we started from the three smallest size distributions. If the collapse criteria for the triplet were obeyed, adjacent larger size distributions were added one at a time to determine if they followed the same criteria. Thus, we identified size windows comprising at least three size distributions, grouping them into windows with isometric (*δ* = 1) or nonisometric (*δ* ≠ 1) scaling behavior. The Anderson–Darling (AD) test was employed to determine whether the *B* distributions of each size window, rescaled according to the exponent for the best collapse, can be effectively considered as belonging to a unique general probability function, as described by Eq. **5**. Only the size windows for which the scaling was nonisometric, Φ < 10%, and the AD null hypothesis accepted were considered as candidates for physiological relevance.

### Evaluation of Robustness and Minimum Number of Spheroids for Consistent Data Collapse.

The robustness of our approach to slight changes in the FE input datasets was assessed through a sensitivity analysis, as detailed in *SI Appendix*, Fig. S4.

We also evaluated the minimum value of the functionals *f*^*res*^(δ) and *f*^*dis*^(δ) against *N* (ranging from 25 up to 10^6^) for a selected size window. This served to identify the minimum number of spheroids required to ensure consistent data collapse, indicative of a rigorous analysis. The selected window—comprising the 9th to the 11th size distributions of stem cell-laden spheroids—fit the criteria of nonisometric scaling, viability, collapse, and *P* > 0.05 for the AD test (*SI Appendix*, Table S5).

### Study of *m*–*B* Joint Distributions.

Extending the analysis to the covariance of *m* and *B*, we coupled the data of spheroid masses with the *B*s derived from the FE models to identify a joint probability density function able to define whether and how size fluctuations may explain or influence the variability of spheroid metabolic rates.

The generalized scaling functional reported in ref. 31 for the joint distributions was hypothesized:

$$p\left(m,B|\langle m\rangle ,\alpha ,\beta \right)=\;m^{-\alpha }B^{-\beta }G\left(\frac{m}{{\langle m\rangle }^{\gamma }},\;\frac{B}{{\langle m\rangle }^{\delta }}\right),$$ [6]

where *α* and *β* are normalization exponents—both kept equal to one as known conditioning parameters, in accordance with ref. 30—*γ* and *δ* are the scaling exponents, and *G* is a general scaling function depending on the marginal probability distributions. As shown in ref. 31, Eq. **6** recovers the traditional allometric relations upon computing marginal distributions and also admits the possibility that the scaling of the means may be affected by the correlated fluctuations in mass and metabolism. Note that, since *m* and *B* are assumed to be mutually dependent variables, their joint probability density function is not equal to the product of the corresponding marginals [i.e., *p*(*m*,*B*|*m*, *α*, *β*) ≠ *p*(*m*|*m*, *α*) × *p*(*B*|*m*, *β*)]. The procedure for reaching the best collapse of joint distributions was based on the functional *g*(*γ*, *δ*) (*SI Appendix*, Eq. **S5**), which is essentially an extension of the distance-based method to the 3D case (see *SI Appendix* for further details).

The optimization process was applied to groups of consecutive spheroid size distributions within the whole range of sizes analyzed using the same rationale employed for the marginals (*SI Appendix*, Fig. S2). However, since a statistical test corresponding to the AD is not available for multivariate distributions, an alternative approach for statistical validation of collapses was used. Specifically, we evaluated how far each rescaled joint distribution within a size window was from a Gaussian by implementing a multivariate normality test (Henze–Zirkler test). If all the size distributions of the window were approximately normally distributed (i.e., significance level of 1%), we performed a three-way ANOVA on the best collapsed distributions for determining whether they are described by the same vector of mean values and, thus, reasonably belong to a unique joint probability distribution (Eq. **6**).

As described for the marginals, we evaluated the minimum number of cell-laden spheroids required for ensuring a consistent minimum of the functional *g*(*γ*, *δ*).

### Probing the Influence of Variability on the Estimation of Scaling Coefficients.

To determine how changes in variability might influence the estimation of *δ*, the distance-based method was used to estimate scaling exponents for joint *m* and *B* distributions with different values of *q*_2_ and sOCR SDs (σsOCR). Following the pipeline summarized in Fig. 1*A* and the methodology for studying joint distributions described above, a subset of spheroids belonging to the size distribution from the 8th to the 10th (*SI Appendix*, Table S2) was generated by using different combinations of *q*_2_ and σsOCR (*SI Appendix*, Table S8).

## Results

### Evaluation of the Minimum Number of Samples Needed for Data Collapse.

The data generated from the FE simulations were used to determine metabolic rate *B* and nonviable volume fraction Φ for each cell-laden spheroid. To identify the minimum number of mass and *B* data points necessary for guaranteeing a consistent collapse, we studied the trends of the optimization functional minima (*SI Appendix*,**S2**, **S4**, and **S5**) against the number of spheroids within each size distribution, *N*. As an example, in Fig. 1, we report the minimum of *f*(*δ*) and *g*(*γ*, *δ*) versus *N* for three consecutive stem cell spheroid size distributions (stem cell-laden spheroids from the 9th to the 11th distribution; *SI Appendix*, Table S2). As expected, the minimum of *f*(*δ*) decreases as *N* increases, reaching a plateau from *N* = 200, using both the residual-based (orange solid line in Fig. 1*B*) and the distance-based method (blue solid line in Fig. 1*B*). A similar trend was obtained for the minimum of *g*(*γ*, *δ*) (Fig. 1*C*), with an expected shift toward a higher number of spheroids necessary for reaching a consistent minimum (from *N* = 10^4^). In the graphs, the peaks of the function minimum obtained for small values of *N* are artifacts due to histogram binning. Specifically, if *N* is not sufficiently higher than the number of bins used, then the histograms coming from the sampling of the initial size distributions (or, in a laboratory context, from carrying out *N* measurements) do not adequately represent the true shape of the corresponding probability density functions.

### Collapse of the Marginal *B* Distributions and Sensitivity Analysis.

**Fig. 2.**
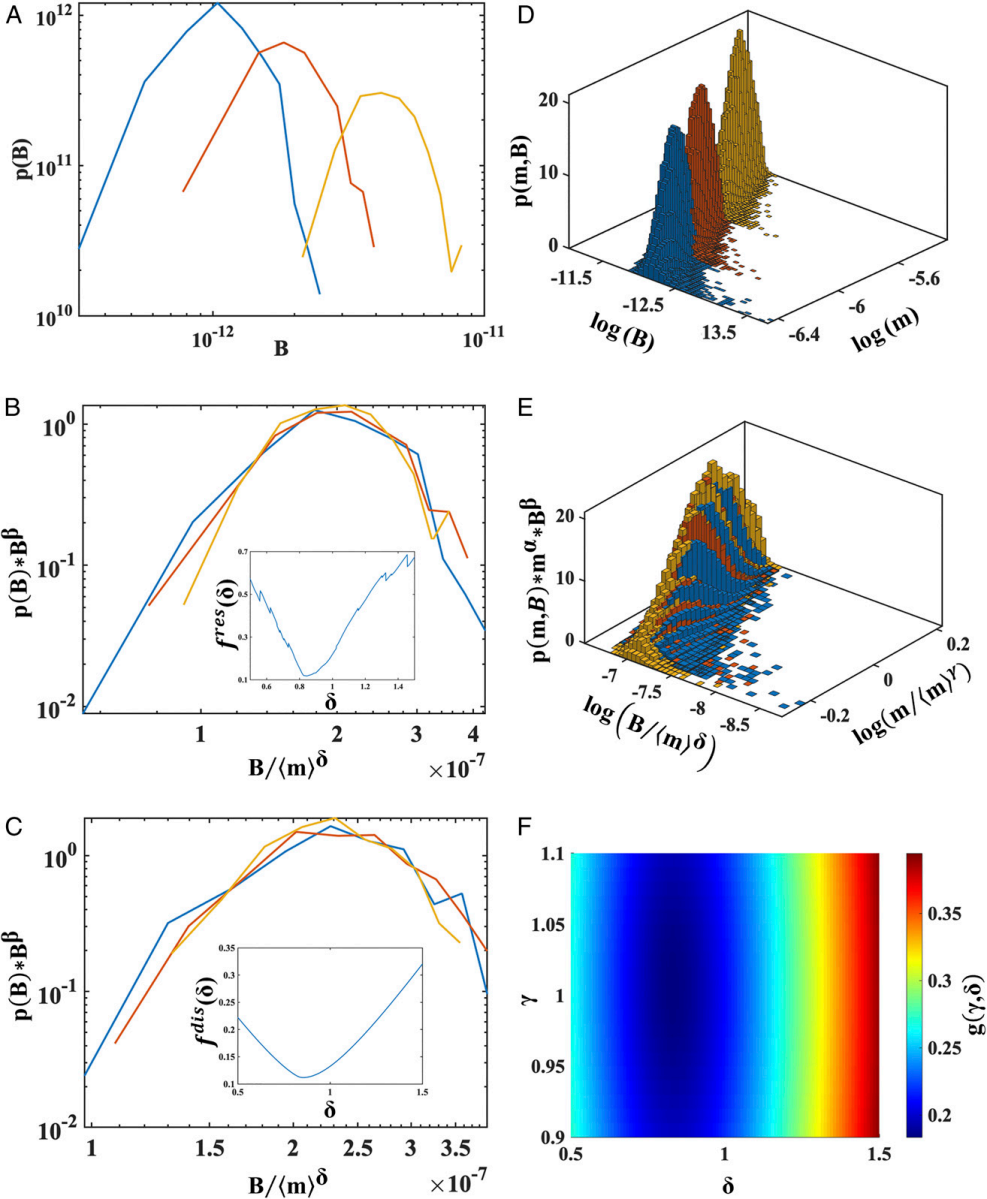
(*A*) An example of a log–log plot of *B* distributions for three stem cell-filled spheroid size distributions (from the 9th to the 11th, *N *= 200 for each size distribution; *SI Appendix*, Table S2). (*B*) Log–log plot of the collapse for the same three subsets, optimized using the residual-based method. (*B*, *Inset*) The functional *f*^*res*^(*δ*) (*SI Appendix*, Eq. **S4**) against *δ*: The minimum of this functional is at *δ* = 0.844 ± 0.008, and the AD test confirms they belong to a common distribution (*P* = 0.295). (*C*) Log–log plot of the collapse for the same three subsets, optimized using the distance-based method. (*C*, *Inset*) the functional *f*^*dis*^(*δ*) (*SI Appendix*, Eq. **S2**) against *δ*: The minimum is at *δ* = 0.83 ± 0.02, and the AD test again confirms they belong to a common distribution (*P* = 0.067). (*D*) An example of joint mass–*B* distributions for three stem cell-laden spheroid subsets (from the 8th to the 10th; *N* = 10^4^ for each size distribution). (*E*) Optimized collapse for the same three subsets. (*F*) Surface plot of *g*(*γ*, *δ*) (*SI Appendix*, Eq. **S5**) against *γ* and *δ*. The minimum of this functional optimizes the collapse of the distributions and corresponds to *γ* = 1.00 ± 0.01 and *δ* = 0.85 ± 0.05. Three-way ANOVA confirms the statistical significance of the collapse (*P* = 0.039). Note that reported ranges of *γ* and *δ* refer to a 1% variation of *f*(*δ*) or *g*(*γ*, *δ*) around the respective minimum.

An example of probability distributions *p*(*B*|⟨*m*⟩, *β*) for a stem cell-laden spheroid size window that fit the criteria of nonisometric scaling, viability, collapse, and *P* > 0.05 for the AD test is shown in Fig. 2*A*. Data collapses of the same rescaled distributions obtained with the residual- and distance-based methods are reported in Fig. 2*B* and *C*, with the respective optimization functionals shown as *Insets*. As detailed in the figure legend, the results from the two approaches are comparable. The values of *δ* resulting from all the statistically significant collapsed size windows derived for both the methods are listed in *SI Appendix*, Table S5 for stem cell spheroids. *SI Appendix*, Table S6 reports the values of *δ* for hepatocyte spheroids using the distance-based method. It should be noted that nonisometric behavior (identified as a value of *δ* significantly different from one) emerges in different size windows for the two cell types because of their different reaction parameters (*SI Appendix*, Table S3).

The robustness of the scaling pipeline depicted in Fig. 1*A* was confirmed by assessing its sensitivity to slight changes in the input dataset of FE models, as shown in *SI Appendix*, Fig. S3.

### Collapse of the Joint *m*–*B* Distributions and Identification of Size Windows for Physiological Relevance.

An example of the joint probability distributions *p*(*m*, *B*|⟨*m*⟩), *α*, *β* of three consecutive stem cell-laden spheroid distributions that fit the criteria of nonisometric scaling, viability, collapse, and *P* > 0.01 for the three-way ANOVA test is shown in Fig. 2*D*.

The collapse of their rescaled distributions is reported in Fig. 2*E*, with the corresponding functional *g*(*γ*, *δ*) in Fig. 2*F*. All the significantly collapsing adjacent joint distributions and the relative values of parameters calculated for stem cell and hepatocyte spheroids are listed in *SI Appendix*, Table S7.

**Fig. 3.**
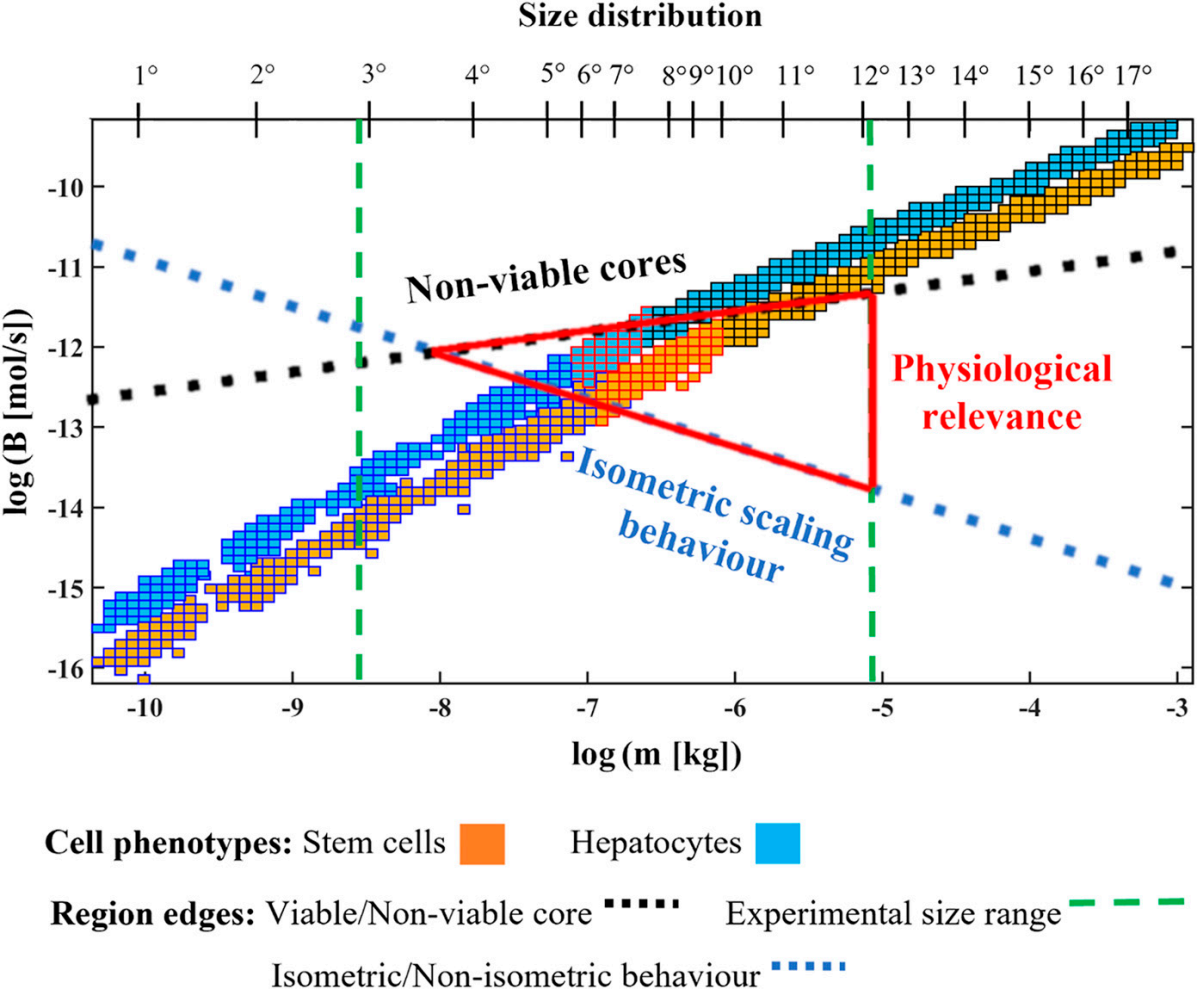
Mass–*B* joint probability distributions for in silico stem cell- and hepatocyte-laden spheroids for all datasets projected onto the log–log mass–*B* plane. The blue dotted line depicts the transition from isometric to nonisometric scaling behavior (estimated using the methods reported in ref. 14). Above the black dotted line, spheroids have a nonviable core due to limits of oxygen diffusion (estimated using the methods reported in refs. 14 and 21). The range of in vitro spheroids reported in the literature lies between the green dashed lines. Following the pipeline in Fig. 1, the intersection of the blue, black, and green lines delimits the physiologically relevant window (full red triangle). The secondary horizontal axis refers to the 17 size distributions with mean radii ⟨*R*⟩ reported in *SI Appendix*, Table S2.

Following the pipeline in Fig. 1*A*, the information on the nonviable core volume obtained from the FE analysis combined with the resulting values of the scaling exponents *γ* and *δ* allows the identification of size windows with nonisometric behavior and an acceptable value of Φ (<10%). These size windows of physiological relevance account for correlated fluctuations of both mass and *B* (Fig. 3).

### The Amplitude of Variability Conditions the Scaling Coefficient.

**Fig. 4.**
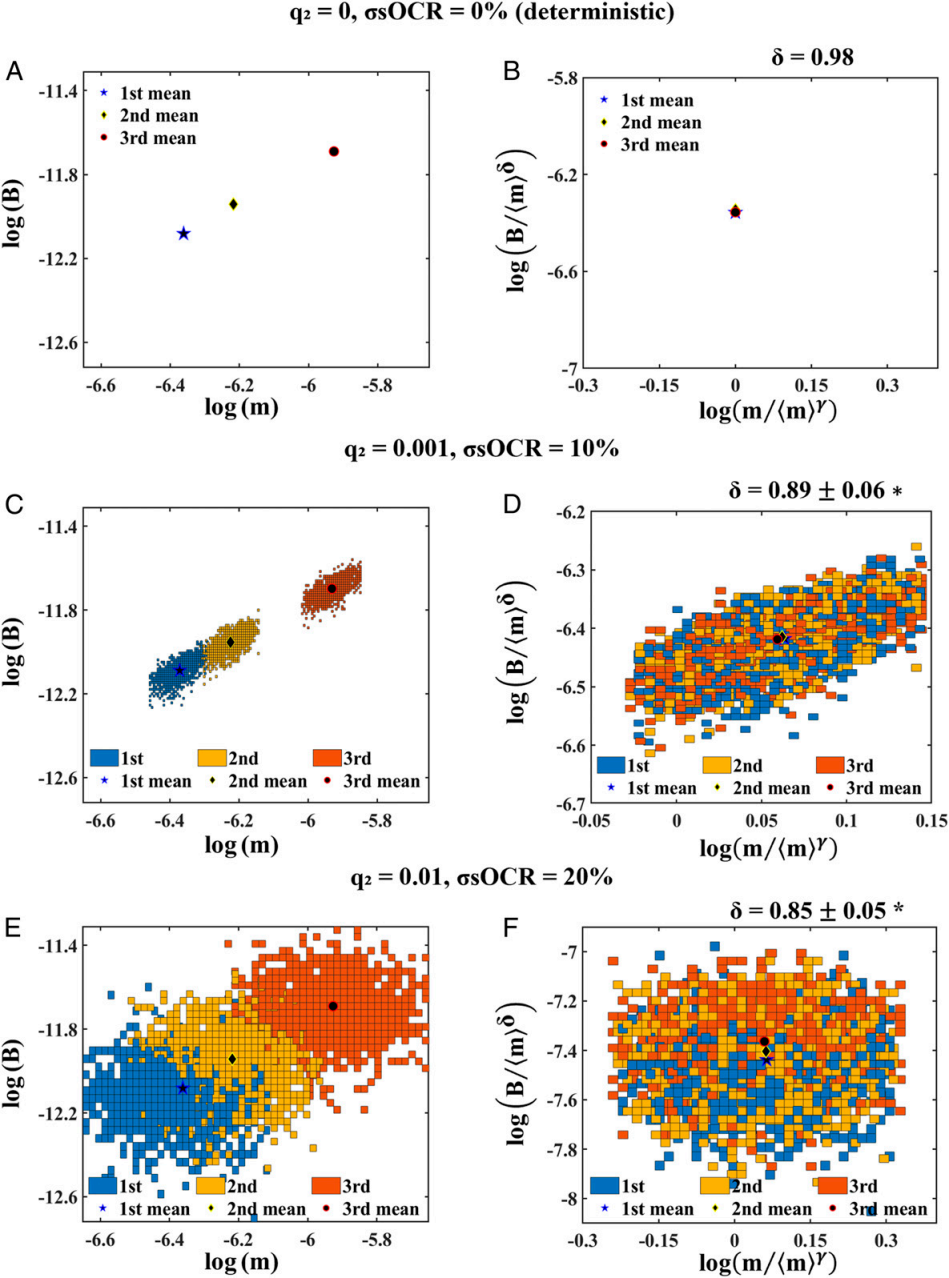
Application of the in silico pipeline to three consecutive *m*–*B* joint distributions (from the 8th to 10th), for spheroids with different values of *q*_2_ and σsOCR. (*A* and *B*) No variability. (*A*) Points corresponding to average (*m*, *B*) values for each size distribution. (*B*) The points in *A* after rescaling and collapse. In the absence of variability, exact values of *γ*(= 1.00) and *δ* are computed. (*C* and *D*) *q*_2_ = 0.001, σsOCR = 10%. (*C*) The *m*–*B* joint distributions and the three points corresponding to the same average (*m*, *B*) values as in (*A*). (*D*) The distributions in *C* after rescaling and collapse. (*E* and *F*) *q*_2_ = 0.01, σsOCR = 20%. (*E*) The *m*–*B* joint distributions and the three points corresponding to the same average (*m*, *B*) values as in (*A*). (*F*) The distributions in *E* after rescaling and collapse. *The range of values for *δ* that best collapse the three rescaled distributions refer to a 1% variation of *g*(*γ*, *δ*) around its minimum. *γ* does not deviate significantly from 1.00.

Fig. 4 shows the application of the in silico pipeline to three significantly collapsed *m*–*B* joint distributions (size distributions from the 8th to 10th) for cell-laden spheroids with different levels of variability and the corresponding values of *δ*. *SI Appendix*, Table S8 summarizes the scaling coefficients obtained for the different combinations of *q*_2_ and σsOCR.

Unremarkably, in Fig. 4*B*, the three average (*m*, *B*) values collapse to a single point, but the points corresponding to rescaled average (*m*, *B*) values do not perfectly overlap if the collapse is optimized with respect to the complete joint distributions (Fig. 4 *D* and *F*). Notably, the outcome of the procedure significantly differs in the three cases: The ranges of *δ* from the collapse of joint distributions do not comprise the exact value obtained from the collapse of the three average (*m*, *B*) values.

## Discussion

Heterogeneity in organismic body mass and fluctuations of metabolic rates (basal, field, or maximal, possibly coexisting in an ecological context) are unavoidable features of any real-life context, including simple (or less than simple) aggregations of cells. Fluctuations of biological parameters with respect to means are inevitable, but largely ignored in current approaches to 3D tissue cultures and scaling. The time thus seems ripe for the development of new experimental and theoretical approaches that incorporate this ubiquitous feature of life, which, like mass–*B* relationships, is present across scales. Characterizing the distributions of features such as size and metabolic rates is an onerous task, particularly in microscaled systems. Therefore, we adopted a computational approach, simulating experimental scenarios to test our hypothesis that fluctuations are important, can help design in vitro experiments, and may also lead to unexpected results.

Following the methodological pipeline shown in Fig. 1*A*, we used FE methods to compute the overall metabolic rate (*B*) and oxygen-concentration profile of randomly generated cell-laden spheroids. Based on our experimental data, spheroids with 17 different mean radii were generated with log-normal distributions of sizes. The individual cells within the spheroids were randomly assigned an intrinsic sOCR from a Gaussian distribution, while the overall volumetric consumption of cells (i.e., *B*) within the spheroids was determined assuming Michaelis–Menten kinetics. A number of generic mass action laws (Monod and Hill) were also used, giving similar outcomes in terms of collapse. Given the importance of stem cells in organoid technology and regenerative medicine and the central role of hepatocytes in body metabolism, two sets of spheroids with mean sOCR and *k*_*M*_ values typical of the two cell types were considered and computationally reproduced. Although impracticable in a laboratory experiment, thanks to the computational pipeline, 1 million spheroids were generated per size distribution and per cell type to enable the identification of the minimum number of samples necessary for achieving robust analyses in subsequent steps.

The *B* data obtained from the FE simulations were rescaled according to Eq. **5**, combining the maximum number of adjacent marginal distributions to achieve a statistically significant collapse. Two different methods were used to determine the value of the exponent *δ* describing the best collapse of up to three rescaled adjacent *B* distributions: Bhattacharjee and Seno’s residual-based method [minimizing *f*^*res*^(*δ*); *SI Appendix*, Eq. **S4**] described in ref. 38 and the purposely developed distance-based method [minimizing *f*^*dis*^(*δ*); *SI Appendix*, Eq. **S2**]. Both methods gave comparable results. Thus, the distance-based method is a valid alternative for assessing the goodness of collapse of 2D probability distributions, and, unlike other methods, it is not limited to 2D distributions, but can be also applied to the analysis of multivariate datasets and *n*-dimensional collapses. A further advantage with respect to the residual-based approach is its lower computational cost. The Anderson–Darling test was employed to determine whether the collapsed datasets are drawn from the same family of distributions.

Given the capability of generating a large number of samples in silico, we were able to define the minimum number of spheroids (*N*) per size distribution required for obtaining an acceptable collapse. For the marginal *B* distributions, a consistent minimum for both *f*^*dis*^(*δ*) and *f*^*res*^(*δ*) was obtained for *N* ≥ 200.

The analysis of oxygen-concentration profiles and *B* marginal distributions enabled the identification of a physiologically relevant size window where the criteria of nonisometric scaling and at least 90% viability were met. According to this definition, the in silico stem cell spheroids are physiologically relevant between the 9th and the 11th size distributions (*δ* = 0.84 ± 0.02), while for hepatocytes, the useful window is from the 9th to 12th size distributions (*δ* = 0.73 ± 0.02). Size windows with smaller radii follow isometric scaling, as they have a *δ* close to one. The size windows are similar to those obtained using deterministic methods for identifying computer-generated spheroids that obey KL (*δ* = 0.75) (14, 21). The deterministic approach differs substantially from the procedure used here: A single mean value of mass and metabolic rate and *δ* = 0.75 are assumed a priori, while our in silico pipeline solves for the best collapse of distributions to identify the most appropriate value of *δ* in the presence of fluctuations.

Having confirmed the validity of the distance-based minimization method, we performed a more exhaustive analysis of covariations in mass and *B*, computing the exponents *δ* and *γ* by minimizing the function *g*(*γ*, *δ*) for up to three rescaled adjacent joint distributions. Three-way ANOVA was applied to test the families of collapsed distributions. Again, we calculated the number of spheroids per joint distribution required to have a consistent estimation of 3D collapse parameters. The number of samples needed (*N* > 10^4^) is two orders of magnitude higher than that estimated for the marginal distributions (*N* = 200). This is expected, as the generalized scaling equation (Eq. **6**) describes the relationship between two covarying aleatory variables (i.e., mass and *B*), rather than just the fluctuations pertaining to a single variable. While *N* = 200 per size range may be feasible for in vitro experiments, higher numbers would be a massive challenge.

Using a parallel approach to that used for the marginals, physiologically relevant size windows for the computer-generated spheroids with joint variations in mass and *B* were identified. In all cases, the mass scaling exponent, *γ*, was, as expected, equal to one. For stem cell spheroids, the useful window lies between the 8th and 10th size distributions (*δ* = 0.85 ± 0.05); for hepatocytes, the window is between the 6th and 8th size distributions (*δ* = 0.81 ± 0.02). The values of *δ* are significantly different from those obtained for the marginal distributions. This is an exact result, underlining the importance of incorporating fluctuations and variability in both size and *B* when estimating metabolic scaling exponents.

Fig. 3 summarizes the results of the application of our in silico pipeline to the design of physiologically relevant in vitro models. From the figure, spheroids that lie in a small mass–*B* window (represented by the red triangle) can be considered as physiologically relevant; all other combinations of *B* and mass either scale isometrically or have an unacceptable high volume of nonviable centers.

Notably, physiologically relevant size windows containing consistently fewer size distributions with smaller mean radii were obtained, evaluating the collapse of joint mass–*B* distributions with respect to the marginals. Experimentally, organoids range from about 100 μm to 3 mm in diameter (36, 39, 40), which suggests that neither the smaller ones nor the larger ones are physiologically relevant.

Biological fluctuations have typically been neglected in the study of cells in culture—indeed, they are currently considered to be irrelevant. Organoids, for example, are well known to manifest a great deal of variability and sensitivity to external perturbations, and much effort is being dedicated to their standardization and reproducibility. That the distributions of size and other physiological parameters are more dispersed as the mass of an organism increases is well known: This feature is characterized by an increase in variance along with size, as outlined in refs. 30 and 41. In this light, the fluctuations observed in organoids and other 3D-engineered tissues are an aspect to be valorized, and, rather than discarded, they might be used in extrapolating physiological features of in vitro models to their in vivo counterparts, so as to encompass the dispersion of size and metabolic rates observed at larger mass scales. On this basis, a statistically significant collapse of joint mass–*B* distributions will be a demonstration that, once rescaled, in vitro and in vivo samples might belong to a unique universal joint probability density function. The implication is that the in vitro models possess the basic elements for translational potential, with impacts in many areas of biomedical science: from reducing animal experiments to regenerative and precision medicine.

We also performed a more generalized analysis, using the pipeline to collapse a subset of distributions of in silico spheroids with different levels of variability in mass and *B* to determine how the amplitude of fluctuations might affect the scaling exponent *δ*. Spheroids with no variability were simply represented by their mean values of mass and metabolic rate, while, given the paucity of information on real fluctuations in microorganisms, intermediate values were chosen arbitrarily. The results reported in Fig. 4 show that for joint mass–*B* probability distributions with increasing variance there is a significant shift of *δ* to lower values (*P* = 0.03). Specifically, *δ* = 0.98 in the absence of fluctuations and decreases from 0.89 ± 0.06 to 0.85 ± 0.05 with increasing mass and *B* variability: There are no overlaps in the values. Again, this is an exact result demonstrating that the presence of covariations in mass and metabolic rate can affect the value of the scaling exponent, and, hence, the method we developed is necessary to deal with biological data that are characterized by intrinsic fluctuations. Should the extent and nature of covariation be confirmed through rigorous measurements in biological systems, our understanding of allometric scaling will need to be rethought, and—to return meaningful results—its applications to the study of pharmacokinetics and dose extrapolation as well as population dynamics and resource utilization will need to take into account covariances in mass and other biological parameters.

In summary, we report an in silico pipeline for analyzing size-dependent scaling in 3D cell-culture systems, taking into account correlated fluctuations in mass and metabolic rates, which are simply unavoidable in nature. The pipeline includes a computationally efficient procedure to assess the goodness of collapse of joint probability density functions and determine the number of data points needed for incorporating fluctuations and covariations in mass and metabolic rate to allow meaningful estimates of scaling exponents. Using in silico spheroids as a test bed for the pipeline, we demonstrate that biological variability contributes to determining scaling exponents and that the exponents may change significantly in the presence of correlated fluctuations.

The framework can aid in the design of in vitro experiments, to identify an optimal range of physiologically relevant cell-aggregate sizes, and to generate interpolative data points for performing exhaustive statistical analyses. Beyond the application proposed here, the in silico pipeline can also be adapted to other types of cell aggregates with different shapes, metabolic requirements, or growth rates, such as microbial communities (42, 43), leading to a better understanding of how scaling emerges in cellular ecosystems.

## Supplementary Material

Supplementary File

## Data Availability

All study data are included in the article and/or *SI Appendix*.
