# Supplementary material for: Scaling of joint mass and metabolism fluctuations in in silico cell-laden spheroids
Source: Proc Natl Acad Sci U S A. 2021 Sep 15;118(38):e2025211118. doi: 10.1073/pnas.2025211118 (PMC8463845; doi:10.1073/pnas.2025211118)
Supplement: Supplementary File [file pnas.2025211118.sapp.pdf]

Supplementary Information for
"Scaling of joint mass and metabolism fluctuations in *in silico* cell-laden spheroids"

Ermes Botte[a,b, +], Francesco Biagini[a,b, +], Chiara Magliaro[a], Andrea Rinaldo[c,d], Amos Maritan[e] and Arti Ahluwalia[a,b, *]

[a] Research Centre "E. Piaggio", University of Pisa
[b] Department of Information Engineering, University of Pisa
[c] Laboratory of Ecohydrology, École Polytechnique Fédérale de Lausanne
[d] Department of Civil, Environmental and Architectural Engineering, University of Padova
[e] Department of Physics and Astronomy "G. Galilei", University of Padova

[+] shared first authorship
* corresponding author: Arti Ahluwalia, Research Centre "E. Piaggio" – University of Pisa, Largo Lucio Lazzarino 1, 56125 Pisa, Italy, +39 0502217062,
Email: arti.ahluwalia@unipi.it

This PDF file includes:

Supplementary text
Figures S1 to S4
Tables S1 to S8
SI References

Supplementary Information Text

Characterization of cell size distributions and extrapolation to cell-laden spheroid size distributions

It has been already shown that micro-organism sizes can be described by a log-normal distribution, whose progressive moments can be computed starting from the mean (1) (main text, Eq. [1]).

The factor of proportionality q_2 - relating the mean values of radius and the corresponding variances of the 3D spheroids - was estimated by assuming that the variability in the size of an organoid or spheroid originates from the variability in the size of the individual cells it is composed of. To this end, we studied the size distribution of two different cell types (*i.e*. HepG2 cells and neural progenitor stem cells (NPSCs)), using a cell counter (Countess II Automated cell counter, Invitrogen). Repeated measurements of cell size distribution for each cell type were carried out and the resulting histograms are reported in Figure S1.

After verifying that the size distribution r_{cell} yields a log-normal form using the Lilliefors test, we calculated its mean and variance. Then, assuming that the volume of each spheroid corresponds to the total volume of cells it contains, the parameters of the radius distribution of spheroids were derived using Eq. [S1]:

$$r_{sph} = \langle r_{sph} \rangle * r_{cell} * \sqrt[3]{\rho_{cell} * \frac{4}{3}\pi} \qquad \text{[S1]}$$

where r_{sph} and r_{cell} are the distributions of the radii of spheroids and cells, respectively, $<r_{sph}>$ is the average spheroid radius and ρ_{cell} is the cell density.

Eq. [S1] provides a theoretical size distribution of spheroids laden with a sample of cells randomly extracted from the experimentally measured cell size distribution. A log-normal function was fitted to such a r_{sph} distribution for each examined phenotype, and the corresponding values of q_2 were determined from its mean and variance according to Eq. [1] (specifically, $q_2 = \langle R^2 \rangle / \langle R \rangle^2 = \mathrm{variance}/\mathrm{mean}^2$). The results obtained are reported in Table S1. The mean values for the 17 size distributions considered are in Table S2.

Collapse of B marginal distributions

Here we detail the methodology implemented for the collapse of B marginal distributions.

First, we built the relative frequency histogram of the Bs for each size distribution (*i.e.,* the 17 groups of N spheroids) to extract a discrete probability distribution. A coherent binning criterion (*i.e.,* 10 bins) was adopted across the 17 groups and the probability values were computed dividing the relative frequencies by the bin width (*i.e.,* imposing that $\int_0^\infty p(B|\langle m \rangle, \beta)\, dB = 1$). Thus, the bin midpoints were considered as the distribution coordinates on the B axis.

Then, we estimated the value of the exponent δ in Eq. [5] (within the range given in Table S4) that optimizes the collapse of the discrete distributions through an iterative process. For this, a functional $f^{dis}(\delta)$ was defined:

$$f^{dis}(\delta) = \sum_{i=1}^{n-1} \sum_{j=i+1}^{n} \left\{ \sum_{k=1}^{M} \sqrt{\left[\left(X_{i,k}(\delta) - X_{j,k}(\delta) \right)^2 + \left(Y_{i,k} - Y_{j,k} \right)^2 \right]} \right\} \qquad \text{[S2]}$$

where n denotes the number of size distributions involved, M is the number of bins in each histogram (*i.e.* the number of points of each discrete distribution), while $X_{i,k}(\delta) = B_{i,k}/\langle m \rangle_i^{\delta}$,

$X_{j,k}(\delta) = B_{j,k}/\langle m\rangle_j^{\ \delta}$, $Y_{i,k} = B_{i,k}{}^{\beta} p_i\left(B_{i,k}\middle|\langle m\rangle_i, \beta\right)$ and $Y_{j,k} = B_{j,k}{}^{\beta} p_j\left(B_{j,k}\middle|\langle m\rangle_j, \beta\right)$ are the rescaled coordinates of points. From a geometric point of view, $f^{dis}(\delta)$ represents the overall sum of the Euclidean distances between corresponding points of each distribution pair expressed in rescaled coordinates (*distance-based* method). Therefore, the local minimum of $f^{dis}(\delta)$ implies the best data collapse. Specifically, we identified the range of δ corresponding to a variation of 1% around the minimum value of $f^{dis}(\delta)$ by evaluating the following minimization:

$$\arg\min_{\delta} f^{dis}(\delta) \qquad \text{[S3]}$$

The value of δ optimizing the collapse was also estimated by implementing the *residual-based* method, established by Bhattacharjee and Seno (2), as a control. In this case, the functional to be iteratively minimized is:

$$f^{res}(\delta) = \sum_{i=1}^{n-1} \sum_{j=i+1}^{n} \left\{ \sum_{k=1}^{M} \left| Y_{i,k}(\delta) - \tilde{Y}_{j,k}(\delta) \right| \right\} \qquad \text{[S4]}$$

Eq. [S4] quantifies the overall sum of the residuals within pairs of overlapping points for each pair of distributions expressed in rescaled coordinates. Since the X coordinate must be the same within the couple to consistently compute the residual, a distribution from each pair was interpolated using a spline function and the points of the other distribution as query points ($\tilde{Y}_{j,k}$ is the rescaled vertical coordinate for the j-th distribution interpolated with respect to the k-th point of the i-th distribution).

Comparison of collapsing approaches

The *residual-based* method represents a milestone in statistical physics which laid the foundations for the development of many other metrics for quantifying the statistical distance between probability distributions (*e.g.* Hellinger's distance) (3). However, since they leverage on the concept of probability contiguity, all of these metrics involve algebraic operations on colinear points (Figure S3), and thus require an interpolation step to be applied to discrete distributions, which are characteristic of experimental datasets. Interpolation is a computationally expensive procedure, whose accuracy strongly depends on the arbitrarily chosen interpolating function as well as on the number of available query points. Consequently, it is particularly challenging for approximating surfaces in a 3D space, starting from a joint frequency histogram.

On the other hand, as illustrated in Figure S3, our purposely developed *distance-based* metric is directly computed simply coupling corresponding points of distributions from the generated/measured dataset while maintaining triangular inequality – a fundamental property common to all the above-mentioned statistical distances. Since the two methods result in comparable outcomes for collapsing B marginal distributions, the *distance-based* metric is more suitable for extending the collapsing procedure to the 3D case. Using our metrics, we were able to reduce the complexity of the algorithm and improve the accuracy and reproducibility of joint m-B distribution collapses.

Sensitivity analysis

We tested the sensitivity of the pipeline with respect to slight changes in the FE input datasets (*i.e.* distributions of spheroid radii and sOCRs), as a benchmark of robustness. Specifically, selecting 5 adjacent size distributions, we evaluated the collapse of marginals for different randomly and independently extracted samples of both radii and sOCRs (as described in the main text). Then, we compared the estimated ranges of δ. No relevant differences were found varying the input datasets (Figure S4).

Collapse of the m-B joint distributions

If the average of a given random variable (e.g. B) depends on the average of another random variable (e.g. m) then they must have a non-trivial joint probability distribution, i.e. they are not independent. Furthermore, if scaling holds between the two mentioned averages, then a scaling equation, like the one proposed in our manuscript Eq. [6], is the most plausible constraint to impose on the joint probability distribution: it recovers the traditional allometric relations upon computing marginal distributions, admits the possibility that the scaling of the means may be affected by the correlated fluctuations in mass and metabolism and allows prediction of the scaling between successive moments of mass and metabolic rate that could not be deducible otherwise.

In the randomly generated spheroids, the dependency between mass and B is through Eqs. [2] and [4] in the main text. Here we describe the extension of the collapsing procedure to the case of joint m-B distributions described by Eq. [6].

We first extracted a discrete mass-B joint distribution for each of the 17 size distributions of spheroids from the 3D relative frequency histograms (with 10 bins for both mass and B axes), assuming a normalization criterion coherent with the one used for $p(MR|\langle m\rangle,\beta)$ (*i.e.* $\alpha = 1$ and $\beta = 1$). Successively, the midpoint coordinates of square bins were computed, considering them as projections onto the m-B plane of the points from each discrete distribution.

As the *residual-based* and the *distance-based* methods used for the marginal distribution collapse return comparable results, and, as discussed in the previous section, the *distance-based* method is more efficient and extendible to multivariate distributions, we evaluated the best collapse defining a functional analogous to Eq. [S2] for a 3D logarithmic space:

$$g(\gamma,\delta) = \sum_{i=1}^{n-1}\sum_{j=i+1}^{n}\left\{\sum_{k=1}^{M}\sqrt{\left[\left(X_{i,k}(\gamma) - X_{j,k}(\gamma)\right)^2 + \left(Y_{i,k}(\delta) - Y_{j,k}(\delta)\right)^2 + \left(Z_{i,k} - Z_{j,k}\right)^2\right]}\right\} \qquad \text{[S5]}$$

where *n* is the number of involved joint distributions, *M* denotes the number of square bins in each 3D histogram (*i.e.* the number of points of each discrete distribution), while $X_{i,k}(\gamma) = m_{i,k}/\langle m\rangle_i{}^{\gamma}$, $X_{j,k}(\gamma) = m_{j,k}/\langle m\rangle_j{}^{\gamma}$, $Y_{i,k}(\delta) = B_{i,k}/\langle m\rangle_i{}^{\delta}$, $Y_{j,k}(\delta) = B_{j,k}/\langle m\rangle_j{}^{\delta}$, $Z_{i,k} = m_{i,k}{}^{\alpha}B_{i,k}{}^{\beta}p_i\left(m_{i,k},B_{i,k}\middle|\langle m\rangle_i,\alpha,\beta\right)$ and $Z_{j,k} = m_{j,k}{}^{\alpha}B_{j,k}{}^{\beta}p_j\left(m_{j,k},B_{j,k}\middle|\langle m\rangle_j,\alpha,\beta\right)$ are the rescaled coordinates of points in the 3D space. From a geometrical point of view, $g(\gamma,\delta)$ represents the extension of the functional $f^{dis}(\delta)$ to a 3D coordinate system. $g(\gamma,\delta)$ was minimized (according to Eq. [S6]) for every combination of δ and γ (see Table S4), to determine the values of δ and γ corresponding to the best collapse.

$$\arg\min_{\gamma,\delta} g(\gamma,\delta) \qquad \text{[S6]}$$

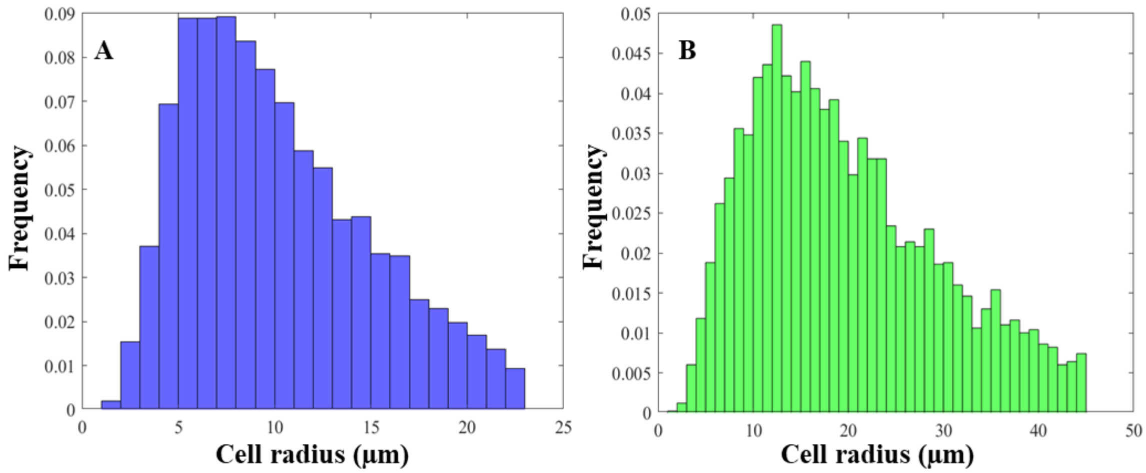

Figure S1. Histograms of the overall experimental distributions of cell radii for **A)** HepG2 cells (mean radius of 11.4 µm) and **B)** NPSCs (mean radius of 24.1 µm). Both the distributions are expressed in terms of relative frequencies.

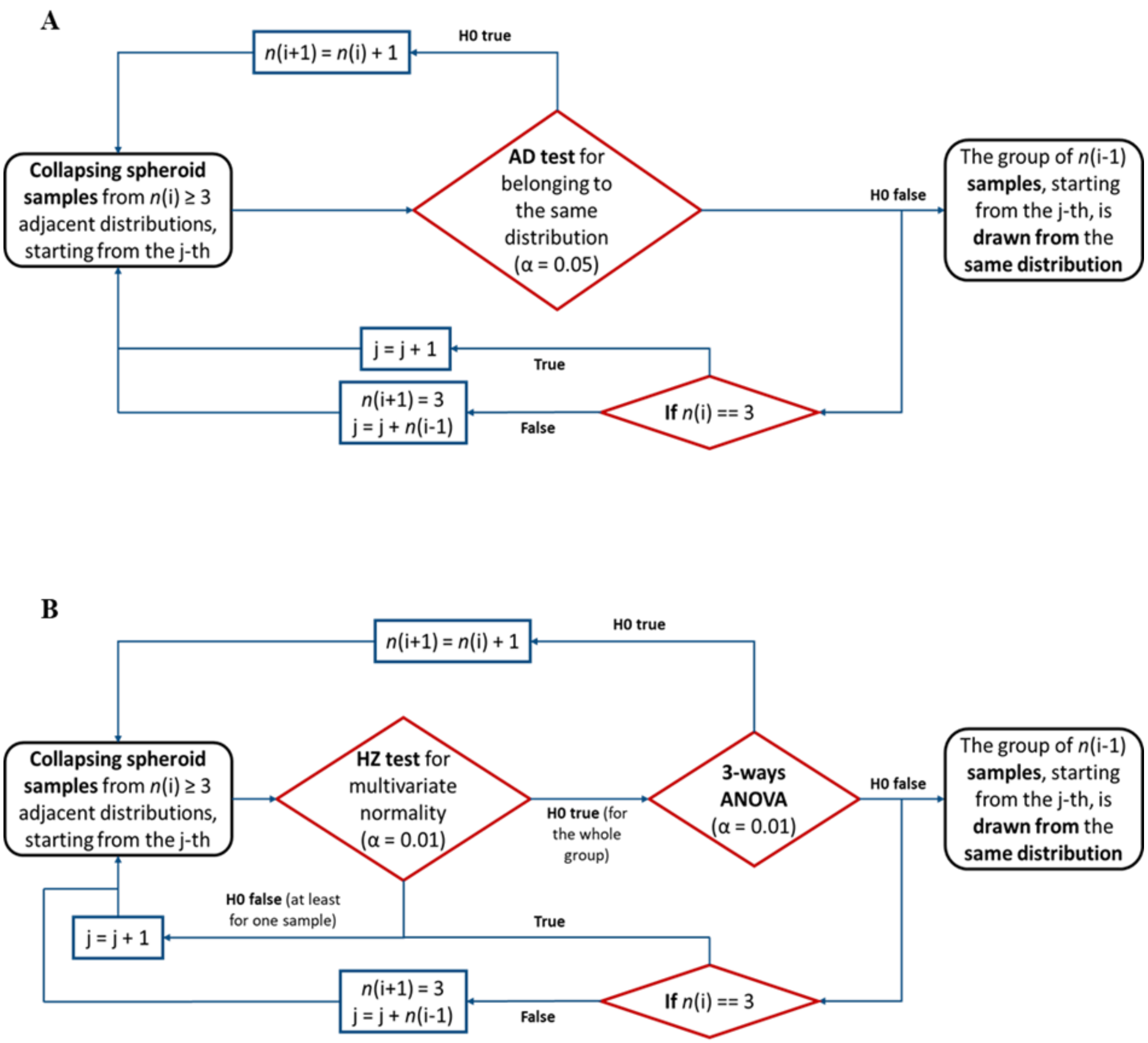

Figure S2. Block diagram of the rationale guiding the statistical analysis of collapses for **A)** marginal B distributions and **B)** joint mass-B distributions. In both cases, *i* denotes temporal iterations of the process and *j* indexes the first of the *n* collapsing distributions.

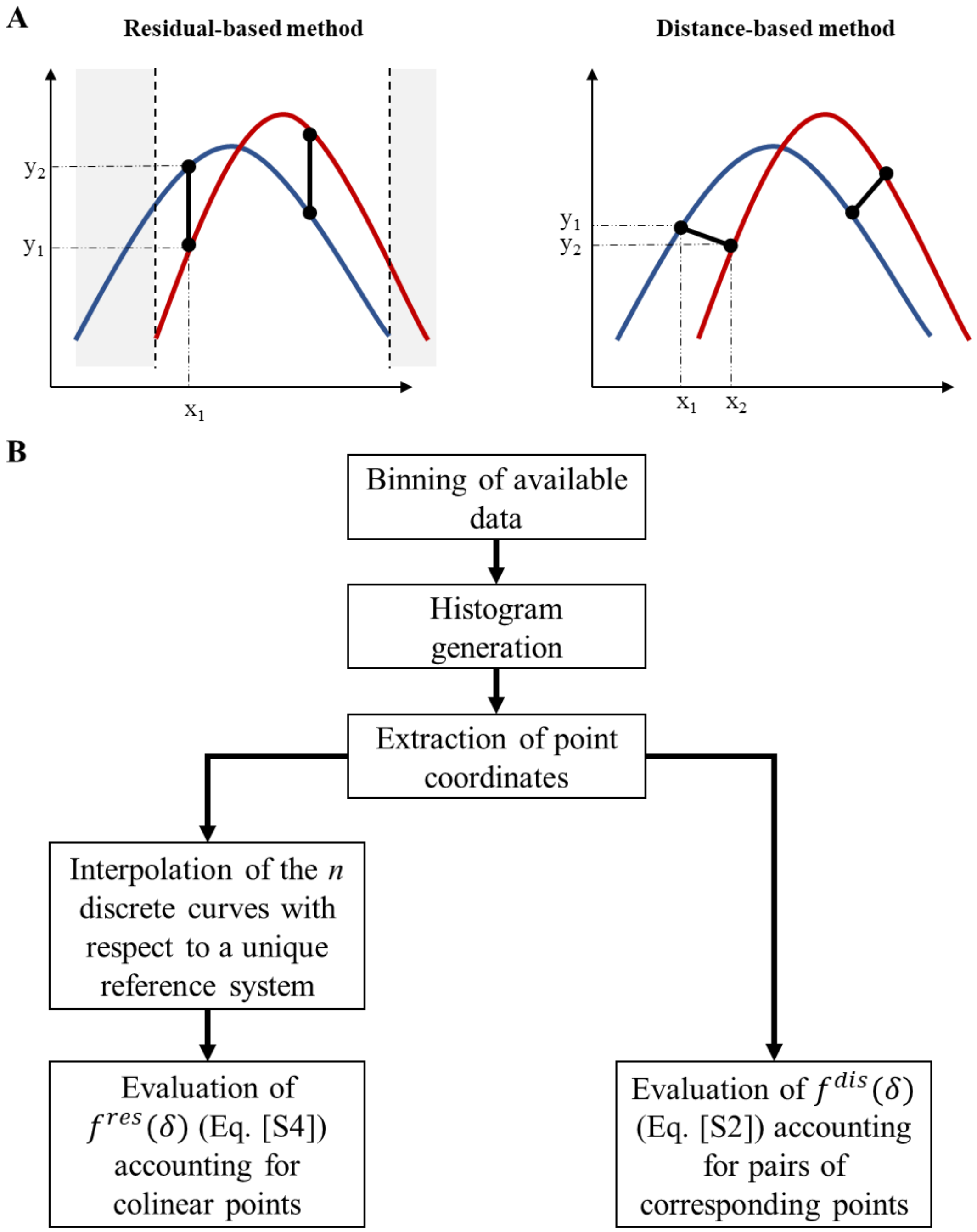

Figure S3. Comparison between the two collapsing approaches implemented. **A)** Graphical representation of the analytical operating principle of both metrics (for the sake of clarity, only the case of marginal distributions is considered, and only two distributions are shown). **B)** Block diagram of the computational pipeline for implementing the collapse of marginal distributions, according to the method applied.

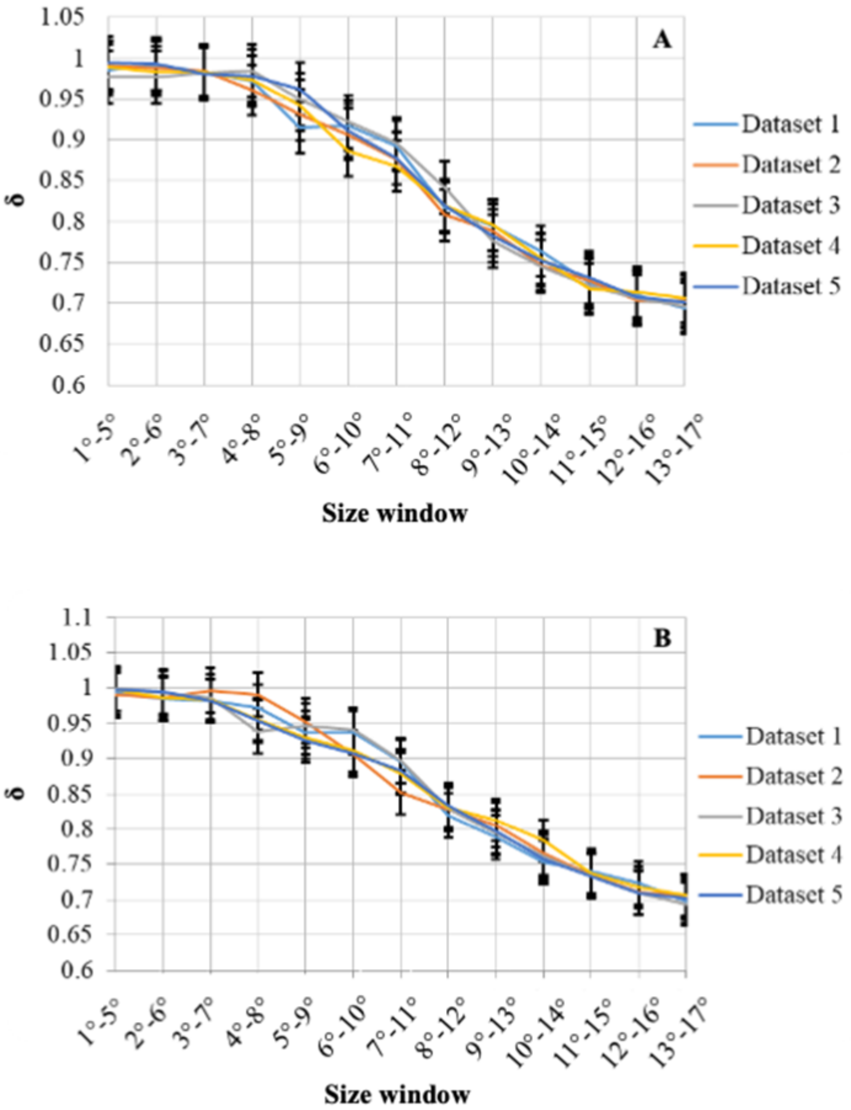

Figure S4. An example of sensitivity analysis to slight changes in the input datasets across the whole range of spheroid radii, considering size windows comprising of five marginal B distributions. **A)** Ranges of δ (corresponding to a 1% variation of $f^{res}(\delta)$ around its minimum) against size windows obtained by using the *residual-based* method of collapse. **B)** Ranges of δ (corresponding to a 1% variation of $f^{dis}(\delta)$ around its minimum) against size windows obtained by using the *distance-based* method of collapse. See Table S1 for matching size window indices and ranges of radii.

Table S1. q_2 derived for spheroids with the two examined cell types, starting from the mean radius of each of the 17 size distributions (see Table S2). q_2 *is* expressed as the median of the 17 values obtained and accounts for the experimentally measured size distributions.

Cell phenotype	Number of cells counted	q_2
HepG2 cells	1.83×10^5	0.0129
NPSCs	3.29×10^5	0.0221

Table S2. Mean values of radii for each of the 17 log-normal size distributions.

Size distribution	Mean spheroid radius, $\langle R \rangle$ (μm)
1st	31
2nd	54.5
3rd	95.8
4th	168.6
5th	236.65
6th	296.6
7th	332.12
8th	466.12
9th	521.8
10th	654.18
11th	918.1
12th	1217.8
13th	1615.3
14th	2142.5
15th	2841.9
16th	3769.6
17th	5000

Table S3. Parameters used for the FE simulations.

Parameter	Range/Values	Notes/Reference
Average values of spheroid radii, $\langle R \rangle$	31 - 5000 μm	17 values as in (4)
Average cell density within spheroids, ρ_c	2.52×10^{14} cell m^{-3}	Similar to *in vivo* (5)
Diffusion coefficient of oxygen within spheroids, D	1.07×10^{-9} m^2 s^{-1}	Typical *in vivo* value (6)
Environmental oxygen concentration	0.21 mol m^{-3}	Henry's law (7)
Average value of sOCR, $\langle sOCR \rangle$	i) 2.75×10^{-17} mol s^{-1} $cell^{-1}$<br>ii) 4.80×10^{-17} mol s^{-1} $cell^{-1}$	The maximum oxygen uptake rate of a single cell. Data for i) stem cells (7) and ii) hepatocytes (4)
Michaelis-Menten constant, k_M	i) 0.201 mol m^{-3}<br>ii) 7.39×10^{-3} mol m^{-3}	Data for i) stem cells (8) and hepatocytes (4)
Critical oxygen concentration for cell viability	0.04 mol m^{-3}	Derived from experiments on brain organoids (9, 10)

Table S4. Range of scaling parameters considered for identifying the best collapse of the B marginal distributions and the mass-B joint distributions.

Parameters		Range/ Values	Notes
**2D B marginal distribution collapse**	Normalization exponent (β)	1	-
	Scaling exponent (δ)	0.5 - 1.5	Values linearly spaced with a step size of 0.001
**3D mass-B joint distribution collapse**	Normalization exponents (α, β)	1	-
	Scaling exponent for mass (γ)	0.9 - 1.1	Values linearly spaced with a step size of 0.01
	Scaling exponent for B (δ)	0.5 - 1.5	Values linearly spaced with a step size of 0.01

For tables S5-S7, physiologically relevant size windows (*i.e*. range of spheroid sizes where both non-isometric scaling and a non-viable volume <10% of the total volume) are in bold.

Table S5. Ranges of δ (considering a 1% variation of $f(\delta)$ around its minimum), corresponding ranges of sizes and non-viable volume percentage (Φ) for stem cell-filled spheroids, evaluated collapsing the marginal probability distributions (*i.e.* $p(B|\langle m\rangle,\beta)$) with both the *residual-based* and the *distance-based* method. Only statistically significant collapses are reported (significance level for the AD test set at 0.05).

**RESIDUAL-BASED METHOD**					
**Spheroid subsets**	**R_{MIN} (µm)**	**R_{MAX} (µm)**	**δ**	**AD test p-value**	**Φ (%)**
**1st-7th**	31	332.12	1.00 ± 0.00	0.599	0
**9th-11th**	**521.8**	**918.1**	**0.84 ± 0.01**	**0.295**	**1.65**
**12th-17th**	1217.8	5000	0.71 ± 0.00	0.055	38.46

**DISTANCE-BASED METHOD**					
**Spheroid subsets**	**R_{MIN} (µm)**	**R_{MAX} (µm)**	**δ**	**AD test p-value**	**Φ (%)**
**1st-7th**	31	332.12	0.99 ± 0.01	0.310	0
**9th-11th**	**521.8**	**918.1**	**0.84 ± 0.02**	**0.067**	**1.65**
**12th-17th**	1217.8	5000	0.69 ± 0.01	0.099	38.46

Table S6. Ranges of δ (considering a 1% variation of $f^{dis}(\delta)$ around its minimum), corresponding ranges of sizes and non-viable volume percentage (Φ) for hepatocyte-filled spheroids, evaluated collapsing the marginal probability distributions (*i.e.,* $p(B|\langle m\rangle,\beta)$) with the *distance-based* method. Only statistically significant collapses are reported (significance level for the AD test set at 0.05).

**Spheroid subsets**	**R_{MIN} (µm)**	**R_{MAX} (µm)**	**δ**	**AD test p-value**	**Φ (%)**
1st-7th	31	332.12	1.00 ± 0.00	0.065	0
9th-12th	521.8	1217.8	0.73 ± 0.02	0.054	48.4
13th-17th	1615.3	5000	0.70 ± 0.01	0.08	82.7

Table S7. Ranges of γ, δ (considering a 1% variation of $g(\gamma,\delta)$ around its minimum), corresponding ranges of sizes and non-viable volume percentage (Φ) for spheroids containing stem cells or hepatocytes, evaluated collapsing the joint probability distributions (*i.e.* $p(m,B|\langle m\rangle,\alpha,\beta)$) with the *distance-based* method. Only statistically significant collapses are reported (significance level set at 0.01).

**STEM CELLS**						
**Spheroid subsets**	**R_{MIN} (µm)**	**R_{MAX} (µm)**	**γ**	**δ**	**ANOVA-3 p-value**	**Φ (%)**
**1st -7th**	31	332.12	1.00 ± 0.01	0.98 ± 0.05	0.0311	0
**8th -10th**	**466.12**	**654.18**	**1.00 ± 0.01**	**0.85 ± 0.05**	**0.039**	**0**

**HEPATOCYTES**						
**Spheroid subsets**	**R_{MIN} (µm)**	**R_{MAX} (µm)**	**γ**	**δ**	**ANOVA-3 p-value**	**Φ (%)**
**1st-5th**	31	236.65	1.00 ± 0.00	0.99 ± 0.01	0.065	0
**6th-8th**	**296.6**	**466.12**	**1.00 ± 0.03**	**0.81 ± 0.02**	**0.058**	**7.2**

Table S8. Ranges of δ (considering a 1% variation of $g(\gamma,\delta)$ around its minimum) for cell-laden spheriods, evaluated collapsing the joint probability distributions (*i.e.,* $p(m,B|\langle m\rangle,\alpha,\beta)$) with the *distance-based* method, considering different combinations of q_2 and σsOCR around the mean sOCR for stem cells. The size window comprising spheroid subsets 8 to 10 (see Tables S2 and S7) was analyzed, and the decreasing trend of δ with increasing amplitude of both mass and B fluctuations was statistically verified (p-value = 0.0311). Since they are always equal to 1, corresponding values of γ are not shown. Double slashes (//) denote combinations of marginal variability (*i.e.,* either the spheroid radius or the sOCR is a deterministic value), which were not evaluated.

	**σsOCR (% of mean)**		
**q_2**	0	10	20
0	0.98	//	//
0.001	//	0.89 ± 0.06	0.86 ± 0.06
0.01	//	0.89 ± 0.09	0.85 ± 0.05

SI References

1. A. Giometto, F. Altermatt, F. Carrara, A. Maritan, A. Rinaldo, Scaling body size fluctuations. *Proc. Natl. Acad. Sci. U. S. A.* **110**, 4646–50 (2013).
2. S. M. Bhattacharjee, F. Seno, A measure of data collapse for scaling. *J. Phys. A. Math. Gen.* **34**, 6375 (2001).
3. L. Le Cam, G. Lo Yang, *Asymptotics in Statistics* (Springer New York, 2000) https:/doi.org/10.1007/978-1-4612-1166-2.
4. A. Ahluwalia, Allometric scaling in-vitro. *Sci. Rep.* **7**, 1–7 (2017).
5. C. Magliaro, A. Rinaldo, A. Ahluwalia, Allometric Scaling of physiologically-relevant organoids. *Sci. Rep.* **9**, 1–8 (2019).
6. F. Caligara, G. Rooth, Measurement of the Oxygen Diffusion Coefficient in the Subcutis of Man. *Acta Physiol. Scand.* **53**, 114–127 (1961).
7. A. Carreau, B. El Hafny-Rahbi, A. Matejuk, C. Grillon, C. Kieda, Why is the partial oxygen pressure of human tissues a crucial parameter? Small molecules and hypoxia. *J. Cell. Mol. Med.* **15**, 1239–1253 (2011).
8. E. Curcio, *et al.*, Kinetics of oxygen uptake by cells potentially used in a tissue engineered trachea. *Biomaterials* **35**, 6829–6837 (2014).
9. M. I. Townsley, R. K. McMillon, A. K. Lee, Regulation of tissue oxygenation in *Seminars in Respiratory and Critical Care Medicine*, (1995), pp. 361–371.
10. E. Berger, *et al.*, Millifluidic culture improves human midbrain organoid vitality and differentiation. *Lab Chip* **18**, 3172–3183 (2018).
